# Reward Sensitivity, Pubertal Development, and Circadian Rhythms: A Window into Adolescent Risk for Depressive Symptoms

**DOI:** 10.1007/s10964-025-02224-6

**Published:** 2025-08-20

**Authors:** Mackenzie A. Maddox, Rachel F. L. Walsh, Logan T. Smith, Thomas Olino, Phyllis C. Zee, Robin Nusslock, Lauren B. Alloy

**Affiliations:** 1 Department of Psychology and Neuroscience, Temple University, Philadelphia, PA USA; 2 Feinberg School of Medicine, Department of Neurology, Northwestern University, Evanston, IL USA; 3 Department of Psychology, Northwestern University, Evanston, IL USA

**Keywords:** Reward sensitivity, Circadian rhythms, Adolescence, Mood disorders

## Abstract

Pubertal maturation is linked to changes in the reward and circadian systems, which may increase vulnerability to psychopathology. Less is known about the associations between reward and circadian systems preceding onset of psychopathology. The current study examined associations between trait reward sensitivity, circadian rhythms, pubertal development, and depressive symptoms. Participants (*n* = 320) were recruited from the Philadelphia area and included 57.5% Females with an average age of 15.12 (SD: 1.02). Participants completed self-reports and one week of actigraphy. Adolescents with higher reward sensitivity and greater pubertal maturity showed greater circadian rhythm disruption. Circadian disruption in combination with reward sensitivity levels predicted differential changes in depressive symptoms, potentially contributing to the understanding of mental health difficulties in adolescence.

## Introduction

Adolescence is an important developmental period when individuals undergo prominent changes in their physical, and often, emotional health (Albert & Steinberg, 2011), including maturation of their reward systems (Forbes et al., 2010), circadian rhythms (Feinberg & Campbell, 2010; Hagenauer et al., 2009), and pubertal development (Holm et al., 2009). Given the multitude of biological, psychological, and social changes that occur, adolescence often demarks an age of vulnerability for psychopathology, including onset of mood disorders (i.e., ages 15–19; Alloy et al., 2012; Kessler et al., 2005; Nusslock & Frank, 2011). Early onset mood disorders have been related consistently to worse prognoses including increases in suicidality and comorbidity (Cirone et al., 2021; Moor et al., 2012), further highlighting the importance of understanding factors that contribute to the increased risk during this period. Although extant work has detailed links between the reward and circadian systems, less work has examined such associations preceding onset of mood disorders during adolescence. Therefore, the overarching goal of the current study was to examine concurrent and prospective associations between trait reward sensitivity, pubertal development, circadian rhythms, and depressive symptoms in a community sample of adolescents to detect potential markers of mood vulnerability.

### Pubertal Development, Reward, and Circadian Rhythms

Adolescence is a time for sensation seeking, risk-taking, and exploration (Steinberg, 2008). Many of these behaviors are associated with puberty-induced changes to the reward system (Braams et al., 2015; Forbes et al., 2010; 2012), mediated by dopamine (Albert & Steinberg, 2011; Casey, 2015). As such, adolescents in mid-to-late pubertal stages tend to display heightened reward sensitivity (Forbes et al., 2010). Although these shifts in reward-related decision making and reward sensitivity are developmentally normative (Casey et al., 2008), elevated reward sensitivity is associated with negative consequences for adolescents, including risky behaviors (e.g., Eaton et al., 2006) and onset of mood disorders (Alloy et al., 2012; Kessler et al., 2005; Nusslock & Alloy, 2017).

Circadian functioning also changes during puberty. Specifically, rest-activity patterns often become disrupted (Crowley et al., 2007; Feinberg & Campbell, 2010), and there is a shift in chronotype (i.e., circadian preference), wherein adolescents gravitate towards activities later in the day (e.g., Hagenauer et al., 2009). Due to social and environmental demands (e.g., early school start times), adolescents often experience circadian misalignment, when their rest-activity rhythm is out of sync with the body’s internal clock, causing sleep disruption and increased depressive symptoms (Smith et al., 2023). In addition, mid- to late-pubertal development is associated with greater sleep disruption compared to pre-/early-pubertal development, often yielding abnormal rest-activity rhythms (Holm et al., 2009). Sleep and circadian dysregulation are independently linked to mental health difficulties (McClung, 2007), and when considered in combination with additional pubertal-induced changes (Forbes et al., 2010; Hagenauer et al., 2009), it is conceptually plausible that more advanced pubertal status may exacerbate the associations between rest-activity rhythms and mental health risk status.

To measure pubertal-induced changes in circadian functioning, researchers often use objective devices such as wearables. Actigraphy data provides objective information regarding individuals’ rest-activity rhythms. Recent statistical developments have yielded non-parametric circadian rhythm indices that are derived from the raw activity counts to better examine rhythmic changes between rest and wake intervals compared to traditional metrics (Blume et al., 2016; Van Someren et al., 1999). Non-parametric circadian rhythm analysis does not prescribe a pre-assumed cosine waveform to the data, thus further allowing a more accurate reflection of an individual’s true circadian rhythmicity (Blume et al., 2016). Although fewer studies have used the non-parametric measures when assessing circadian dysregulation in relation to risk for mood disorders, prior findings suggest patterns related to less robust rhythms, more variability, and less stability (i.e., flattened relative amplitude, a greater intra-daily variability, and a lower inter-daily stability; see Faedda et al., 2016; Jones et al., 2005; Rock et al., 2014).

### Reward-Circadian Rhythm Model

Much work suggests that the reward and circadian systems have bidirectional influences on each other (Alloy et al., 2015; Murray et al., 2009). Specifically, the body’s pacemaker, the suprachiasmatic nucleus (SCN; Grippo et al., 2017), responds to reward-relevant stimuli that act as zeitgebers (i.e., “time-givers”) by entraining the SCN through dopamine release. Consequently, the SCN influences reward-related behavior through neurotransmitter and hormonal release (Dunlap, 1999). These mechanistic connections reflect the interdependence between these two systems. Given the pubertal-induced changes that occur in both the reward and circadian systems, such as the increased sensitivity to rewards and circadian phase delay, it is possible that pubertal maturation further amplifies pre-existing reward-circadian associations in an inherent feedback loop, above and beyond their independent effects (e.g., Hasler et al., 2012; Forbes et al., 2012).

Given that adolescence is a high-risk period for mood disorders such as bipolar spectrum disorder (BSD) (Cirone et al., 2021; Moor et al., 2012), it seemed conceptually appropriate to apply an integrated reward-circadian rhythm theoretical model originally developed from the BSD literature to the current study (Alloy et al., 2015). According to the aforementioned model, individuals with or at risk for developing BSD tend to exhibit increased sensitivity to rewarding stimuli and increased behavioral activation system functioning (BAS; Alloy et al., 2008; 2012; 2016; Alloy & Nusslock, 2019) compared to individuals without or at low risk for BSD. Individuals with BSD or at risk also tend to exhibit dysregulated circadian rhythms as evidenced by irregular rest-activity rhythms (e.g., Murray et al., 2020; McCarthy et al., 2022; Ng et al., 2015; Scott et al., 2021). The above model posits that the reward and circadian systems interact, potentially explaining transitions from trait vulnerabilities (i.e., high trait reward sensitivity) to clinical diagnoses (i.e., BSD). For example, according to the model, when high reward sensitive individuals experience excessive reward system activation or deactivation states, they will engage in behaviors that lead to disregard of normal social routines (e.g., bedtimes, waketimes), which, in turn, disrupt circadian rhythms (Alloy et al., 2015). Together, this model and findings that support the model (e.g., Boland et al., 2016) suggest that higher trait reward sensitivity may contribute to irregular rest-activity rhythms, preceding onset of mood disorder, or more broadly, mood symptoms. Although hypo/manic symptoms are thought of as the more conspicuous features of bipolar mood symptomatology, depressive symptoms often are associated with greater rates of morbidity and mortality (Altshuler et al., 2002; Mitchell & Malhi, 2004). Notably, depressive symptoms (bipolar or unipolar) tend to be prevalent duing early stages, particularly among adolescents (Bowden, 2001). Thus, the reward-circadian model provides a framework to assess potential markers of vulnerability for mood dysregulation during the adolescent period.

## Current Study

Dysregulation of the reward and circadian systems often are found in association with mood dysregulation, and pubertal maturation has been shown to elicit substantial changes in adolescents’ reward sensitivity and circadian phase. However, less is known about how these associations between reward, circadian, and pubertal processes interact before onset of mood disorders. In addition, adolescence is a critical period in which internal, circadian rhythms often don’t align with socially imposed demands such as school. Although not the primary focus, the current study also is motivated by a goal to increase knowledge and awareness surrounding sleep and circadian eduction and its relation to mental health during this important developmental period. Thus, the current study examined concurrent associations between trait reward sensitivity and circadian rhythms, specifically rest-activity rhythms, as well as the moderating impact of pubertal development. As a secondary aim, the current study examined associations between baseline circadian rhythms, specifically rest-activity rhythms, and depressive symptoms at follow-up with trait reward sensitivity as a moderator in an attempt to capture initial signs of mood vulnerability. For Aim 1, it was hypothesized that higher trait reward sensitivity would be significantly associated with greater circadian dysregulation as indicated by a flatter (i.e., lower) relative amplitude, a lower inter-daily stability, and a higher intra-daily variability and that greater pubertal development would amplify these associations. For Aim 2, it was hypothesized that greater circadian dysregulation, as indicated by the same three indices, would be prospectively associated with greater depressive symptoms at follow-up and that higher reward sensitivity would amplify these associations, above and beyond controlling for pubertal development.

## Method

### Participants

Adolescents, ages 13 – 16, were included from two prospective, longitudinal projects investigating predictors of mood disorders at Temple University, in Philadelphia, Pennsylvania: Circadian, Reward, and Emotion Systems in Teens (CREST; for protocol paper, see Alloy et al., 2023) and Reward and Immune Systems in Emotion (RISE; for protocol paper, see Alloy et al., 2023). Both projects recruited participants based on scores on a self-report measure of trait reward sensitivity to assess an underlying reward sensitivity dimension (Liu et al., 2018): Behavioral Activation System (BAS) Scale of the Behavioral Inhibition System/Behavioral Activation System Scales (BIS/BAS; Carver & White, 1994). Both projects were identical in recruitment procedures concerning demographics with slightly varying exclusion criteria (discussed below). Based on the behavioral activation system scale (Alloy et al., 2012), participants were recruited across the entire trait reward sensitivity dimension with greater emphasis on opposing ends of the dimension for each project to better capture at-risk samples.

In CREST, recruitment procedures over-sampled participants on the high dimensional tail of trait reward sensitivity, whereas in RISE, recruitment procedures over-sampled on the low dimensional tail of trait reward sensitivity. Participants were categorized into reward sensitivity quintiles based on the percentile of their behavioral activation system score from over 8000 adolescents screened on the behavioral activation system scale. Participants from all five quintiles were recruited for each project, with the aforementioned over-sampling of the highest or lowest quintile for Projects CREST and RISE, respectively. For the current study, we used data from a subsample of participants across both projects (*n* = 320; 57.5% Female) to include a more complete representation of the full reward sensitivity spectrum: 33.9% quintile 1, 14.9% quintile 2, 13.0% quintile 3, 12.0% quintile 4, and 26.3% quintile 5. The sample was 58.13% Caucasian/White, 28.44% African American/Black, 4.69% Asian, 7.50% Multiracial/biracial, and 1.24% Other race. Of those, 11.04% identified as Hispanic or Latino/a.

Exclusion criteria for CREST included: 1) prior history of DSM-5 BSD diagnosis (i.e., bipolar I, bipolar II, cyclothymia, or bipolar disorder not otherwise specified); 2) a hypomanic episode occurring prior to the date of screening; or 3) consumption of substances or engaged in behaviors known to impact the circadian system, such as melatonin or photosensitizing medication, bright light therapy, shift work, or traveled frequently across time zones. Exclusion criteria for RISE included: 1) prior history of DSM-5 major depressive disorder (MDD) diagnosis; 2) a major depressive episode occurring prior to the date of screening; or 3) autoimmune disease, disorder involving chronic inflammation, or recent history of inflammatory medications usage. Exclusion criteria across both projects included: 1) a diagnosis of any DSM-5 psychotic disorder or current psychosis; 2) inability to complete verbal and written measures administered in English; 3) contraindications for an MRI scan (e.g., metal in the body, claustrophobia, pregnancy); or 4) a medical illness that prevented participation.

### Procedures

At baseline, participants completed self-report reward sensitivity and depression symptom measures and underwent a semi-structured clinical diagnostic interview to assess current and lifetime psychopathology. Immediately following the Time 1 sessions, participants completed a seven-day collection of continuous actigraphy data and corresponding sleep diary questionnaires. Participants were instructed to keep the watches on at all times except for scenarios in which the devices would be immersed in water for prolonged periods (e.g., bathing or swimming) and to complete a sleep diary approximately one hour before bedtime. Approximately six months following Time 1, participants completed a self-report measure of depressive symptoms to assess for change since baseline. All procedures were approved by the Institutional Review Board of Temple University (RISE approval number: 27918; CREST approval number: 28338).

### Measures

#### Reward sensitivity

The Behavioral inhibition system/Behavioral activation system scale (BIS/BAS) is a frequently used, 20-item scale created to capture both behavioral activation and behavioral inhibition (Carver & White, 1994). Participants responded to items on a 4-point Likert scale. For the purposes of this study, responses to the three BAS subscales were assessed: Reward responsiveness, drive, and fun-seeking. The three subscales were summed to create a total BAS score. The BAS consistently displays good re-test reliability, internal consistency, and construct validity with personality traits, emotion, and incentive inspired reaction-time tasks (Colder & O’Connor, 2004; Kambouropoulos & Staiger, 2004; Zinbarg & Mohlman, 1998). The internal consistency for the BAS total score in the current sample was good (*a* = 0.82).

#### Rest-activity rhythms

Participants wore Actiwatch Spectrum devices on their non-dominant wrist for a total of seven consecutive days (Philips Healthcare, Bend, OR). These devices provide a non-invasive, objective method to collect information regarding general sleep and wake intervals and are highly correlated with sleep diaries and polysomnography among healthy and clinical samples (Kaplan et al., 2012; Kushida et al., 2001). Data were collected in 1-minute epochs, and all actograms were manually inspected to accurately determine rest and excluded intervals using activity and white light levels. To allow for a more granular inspection of circadian rhythms, non-parametric circadian indices were derived through the suggested protocols advised by Blume and colleagues (2016).

##### Relative amplitude

Relative amplitude represents the degree to which the circadian rhythm is robust and strong and was calculated by taking the difference between M10 and L5 activity. M10 represents the 10 consecutive, most active hours in a 24-hr period, whereas L5 represents the five consecutive, least active hours in a 24-hr period. M10 and L5 were calculated on a minute-wise level between days to yield a total of 1440 values. Relative amplitude values range from 0 to 1 with higher values more indicative of an individual who exhibits more activity in the daytime interval and more rest in the nighttime interval. The formula for calculation of relative amplitude (RA) is below:

$${RA}=\frac{(M10-L5)}{(M10+L5)}$$

##### Intra-daily variability

Intra-daily variability (IV) quantifies the fragmentation of rest-activity cycles within 24-hr periods. Intra-daily variability values range from zero (Gaussian noise) to two such that it converges to zero for a perfect sine wave. There are cases in which the value can exceed two if a period length of two hours exists within the cycle. Lower values of intra-daily variability are indicative of a healthier circadian rhythm indicating less fragmentation. In the formula below, *n* represents the total sampling points, $\bar{X}$ represents the grand average, and ${X}_{i}$ represents the activity value from each sampling point.

$${IV}=\,\frac{n{\sum }_{i=2}^{n}{({X}_{i}-{X}_{i-1})}^{2}}{(n-1)\,{\sum }_{i=1}^{n}{({X}_{i}-\bar{X})}^{2}}$$

##### Inter-daily stability

Inter-daily stability (IS), in contrast to intra-daily variability, is computed by quantifying the stability of rest-activity rhythms between days. By representing the invariability of the circadian rhythm, inter-daily stability further illustrates the coupling to zeitgebers. Values for inter-daily stability range between 0 and 1, with greater values being indicative of a more stable rhythm with stronger coupling to an external zeitgeber such as light. In the formula below, *n* represents the total sampling points, *p* represents the number of sampling points per day, ${\bar{X}}_{h}$ represents the hourly means, $\bar{X}$ represents the grand average, and ${X}_{i}$ represents the activity value from each sampling point.

$${IS}=\frac{n{\sum }_{h=1}^{p}{({\bar{X}}_{h}-\bar{X})}^{2}}{p\,{\sum }_{i=1}^{p}{({X}_{i}-\bar{X})}^{2}}$$

#### Pubertal development

The Pubertal Development Scale (PDS; Petersen et al., 1988) is a 5-item self-report questionnaire. The PDS includes items pertaining to growth in height and weight, changes in skin, changes in breast and menstruation (females), and changes in facial hair and voice (males). All items excluding menstruation are scored on a 4-point scale ranging from 1 (*no development*) to 4 (*full development*). The menstruation item is scored as either a 1 (*I have not yet begun to menstruate*) or a 4 (*I have begun to menstruate*). Individual items were summed to generate a total score, with a higher score indicating more advanced pubertal development. The PDS displays good reliability (*a* = 0.77; Petersen et al., 1988) and validity in comparison to physicians’ ratings including a moderate correlation to Tanner stages (*r’s* ranging from 0.61 to 0.67; Alloy et al., 2016; Hamilton et al., 2014; Hamlat et al., 2014; Stumper et al., 2019). McDonald’s Omega was used to examine internal reliability given that it often provides a more accurate estimate for multidimensional scales and does not assume tau-equivalence as in Cronbach’s Alpha. The PDS’s internal consistency (ωt) in the current sample was good for males (ωt = 0.79) and lower for females (ωt = 0.67), potentially due to the often-observed variability in the timing of menarche, resulting in less cohesion among items.

#### Depressive symptoms

Participants completed the Beck Depression Inventory-II **(**BDI; Beck et al., 1996) to assess depressive symptom severity at Time 1 and Time 2. The BDI is comprised of 21 items in which participants responded on a scale from 0 to 3 indicating their feelings over the past two weeks. Across a diverse range of samples and age-groups, the BDI demonstrates strong reliability and validity (Osman et al., 2008; Wang & Gorenstein, 2013). The BDI’s internal consistency (*a*) in the current sample was excellent at both time points (T1 *a* = 0.93; T2 *a* = 0.91).

### Data Analysis

All analyses were conducted using R version 4.3.1 (R Core Team, 2023). A simulation-based a-priori power analysis using estimated effect sizes and a sample size of 200 yielded a power of 80% to detect a significant interaction effect at a significance level of 0.05. Due to missing data at the follow-up time point, Aim 2 models have an overall lower sample size in comparison to Aim 1 models. Non-parametric circadian rhythm analysis was conducted using the ‘nparACT’ package (Blume et al., 2016) to produce comprehensive circadian profiles for each participant. Modeling assumptions were evaluated using the ‘performance’ package (Lüdecke et al., 2021).

The main set of analyses involved testing the a-priori main effect and interaction models. For Aim 1, the study examined associations of trait reward sensitivity, pubertal development, and their interaction with the three circadian indices using hierarchical multiple regression, yielding a total of three models. Main effects were included in the first step of each model. The interaction term between trait reward sensitivity and pubertal development was included in the second step of each model. For Aim 2, the study examined prospective associations between the three circadian indices, trait reward sensitivity, and their interaction with Time 2 depressive symptoms using hierarchical multiple regression, yielding a total of three models. Main effects were included in the first step of each model, and the interaction term between the circadian index and trait reward sensitivity was added in the second step. The interaction terms were computed as the product of the centered variables.

Biological sex and race (3-level categorical variable: Caucasian/White, African American/Black, and Other) were included as covariates in all models. Time 1 depression and pubertal development were included as covariates in all prospective models predicting to Time 2 depression. Significant interactions were probed using the Johnson-Neyman Procedure through the ‘interactions’ package (Bauer & Curran, 2005).

## Results

### Preliminary Analyses

All model residuals were approximately normally distributed. Means, standard deviations, and bivariate relationships of key variables are displayed in Table 1. As expected, inter-daily stability was positively correlated with relative amplitude (*r* = 0.85, *p* < 0.01) but negatively correlated with intra-daily variability (*r* = −0.12, *p* < 0.05). None of the three circadian indices were significantly correlated with trait reward sensitivity. Pubertal development was positively correlated with inter-daily stability (*r* = 0.11, *p* < 0.05), Time 1 depression (*r* = 0.20, *p* < 0.05) and Time 2 depression (*r* = 0.15, *p* < 0.05). Intra-daily variability was negatively correlated with Time 1 depression (*r* = −0.11, *p* < 0.05) but not Time 2 depression.

**Table 1:** Descriptive Statistics and Bivariate Relationships of Key Variables

Variable	*M*	*SD*	1	2	3	4	5	6
1. BAS	39.91	5.36						
2. PDS	16.06	2.65	−0.06 [−0.16, 0.05]					
3. RA	0.74	0.22	−0.05 [−0.15, 0.06]	0.10 [−0.01, 0.21]				
4. IV	0.78	0.20	0.04 [−0.07, 0.15]	−0.04 [−0.15, 0.07]	−0.04 [−0.15, 0.07]			
5. IS	0.38	0.17	−0.09 [−0.20, 0.01]	0.11^*^ [0.00, 0.22]	0.85^**^ [0.82, 0.88]	−0.12^*^ [−0.22, −0.01]		
6. BDI.1	6.78	8.31	−0.01 [−0.12, 0.09]	0.20^**^ [0.09, 0.30]	0.08 [−0.03, 0.18]	−0.11^*^ [−0.21, −0.00]	0.10 [−0.01, 0.20]	
7. BDI.2	6.08	6.91	−0.07 [−0.18, 0.04]	0.15^**^ [0.04, 0.26]	0.04 [−0.07, 0.16]	−0.11 [−0.22, 0.01]	0.05 [−0.06, 0.17]	0.63^**^ [0.56, 0.69]

*M* and *SD* are used to represent mean and standard deviation, respectively. Values in square brackets indicate the 95% confidence interval for each correlation. The confidence interval is a plausible range of population correlations that could have caused the sample correlation (Cumming, 2014)

*BAS* behavioral activation scale; *BDI* Beck depression inventory; *IV* intra-daily variability; *IS* inter-daily stability; *PDS* pubertal development scale; *RA* relative amplitude

*p < 0.05. **p < 0.01.

### Aim 1: Cross-sectional Models

Table 2 presents the hierarchical multiple regression results across the three models.

**Table 2:** Aim 1 Regression Results

Hierarchical Multiple Regression						
	*Dependent variable:*					
	RA		IV		IS	
	(1)	(2)	(3)	(4)	(5)	(6)
BAS	−0.002 (0.002)	−0.002 (0.002)	0.002 (0.002)	0.002 (0.002)	−0.003 (0.002)	−0.003 (0.002)
PDS	0.003 (0.005)	0.003 (0.005)	−0.002 (0.005)	−0.002 (0.005)	0.002 (0.004)	0.002 (0.004)
Sex: Female	0.046 (0.029)	0.048 (0.029)	−0.006 (0.027)	−0.005 (0.027)	0.046^*^ (0.023)	0.048^*^ (0.022)
Race^a^: Other	0.003 (0.040)	0.008 (0.039)	−0.026 (0.036)	−0.025 (0.037)	0.022 (0.031)	0.026 (0.030)
Race^a^: White	0.027 (0.028)	0.022 (0.027)	−0.025 (0.025)	−0.026 (0.025)	0.032 (0.021)	0.029 (0.021)
BAS X PDS		−0.003^**^ **(0.001)**		−0.0005 (0.001)		−0.002^**^ **(0.001)**
Constant	0.695^**^ (0.030)	0.695^**^ (0.029)	0.793^**^ (0.027)	0.793^**^ (0.027)	0.329^**^ (0.023)	0.329^**^ (0.023)
Observations	320	320	320	320	318	318
R^2^	0.022	0.048	0.008	0.009	0.041	0.069
Adjusted R^2^	0.007	0.030	−0.008	−0.010	0.026	0.051
Residual Std. Error	0.214 (df = 314)	0.211 (df = 313)	0.196 (df = 314)	0.196 (df = 313)	0.164 (df = 312)	0.162 (df = 311)
F Statistic	1.440 (df = 5; 314)	2.659^*^ (df = 6; 313)	0.477 (df = 5; 314)	0.459 (df = 6; 313)	2.694^*^ (df = 5; 312)	3.836^**^ (df = 6; 311)

*BAS* behavioral activation system scale, *IS* inter-daily stability, *IV* intra-daily variability, *PDS* pubertal development scale, *RA* relative amplitude

*p < 0.05. **p < 0.01.

^a^Reference level for Race: Black/African American

Bold values indicate significant interactions

#### Relative amplitude

**Fig. 1:**
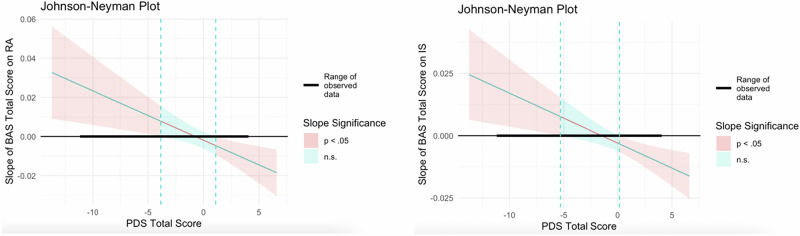
Aim 1 Interactions. The graph on the left panel represents the interaction between BAS reward and pubertal development on relative amplitude whereas the graph on the right panel represents the interaction between BAS reward and pubertal development on inter-daily stability. Predictor and moderator variables were centered for analyses

The first step of the model accounted for 0.69% of the variance in relative amplitude. Trait reward sensitivity and pubertal development were not significantly associated with relative amplitude. In the second step of the model, the inclusion of the interaction term significantly increased the variance accounted for in relative amplitude by 2.34%. The main effects of trait reward sensitivity and pubertal development remained nonsignificant. However, the interaction effect was significant (B = −0.003, *p* < 0.01) (Fig. 1). To better understand the significant interaction, we estimated simple slopes of trait reward sensitivity (BAS) on relative amplitude at regions of significance using the Johnson-Neyman Procedure. The range of observed values of pubertal development was −11.06 to 3.94, and the slope of trait reward sensitivity was significant (p < 0.05) when pubertal development values were outside the interval of −3.85 to 1.11. Specifically, at low levels of pubertal development, trait reward sensitivity was significantly and positively associated with relative amplitude (B = 0.01, *p* < 0.05), whereas, at high levels of pubertal development, trait reward sensitivity was significantly and negatively associated with relative amplitude (B = −0.001, *p* < 0.05). The interaction remained significant after conducting post-hoc sensitivity analyses controlling for age (B = −0.003, *p* < 0.01; see Supplemental Table 1).

#### Intra-daily variability

The first step of the model accounted for less than 1% of the variance explained in intra-daily variability. Neither trait reward sensitivity nor pubertal development were significantly associated with intra-daily variability. The addition of the interaction term in the second step did not significantly increase the variance explained in intra-daily variability by the model. The main effects of trait reward sensitivity and pubertal development remained nonsignificant. The interaction between trait reward sensitivity and pubertal development on intra-daily variability also was not significant.

#### Inter-daily stability

The first step of the model accounted for 2.6% of the variance explained in inter-daily stability. Neither trait reward sensitivity nor pubertal development were significantly associated with inter-daily stability. The second step of the model, which included the interaction term, significantly increased the variance accounted for in inter-daily stability by 2.49%. In this step of the model, the main effects of trait reward sensitivity and pubertal development on inter-daily stability remained nonsignificant. However, the interaction effect between trait reward sensitivity and pubertal development on inter-daily stability was significant (B = −0.002; *p* < 0.01) (Fig. 1).

Per the Johnson-Neyman Procedure, the range of observed values of pubertal development was −11.06 to 3.94, and the slope of trait reward sensitivity was significant (*p* < 0.05) when pubertal development values were outside the interval of −5.31 to 0.16. At very low levels of pubertal development, trait reward sensitivity was significantly and positively associated with inter-daily stability (B = 0.01, *p* < 0.05), whereas, at moderately high levels of pubertal development trait reward sensitivity was significantly and negatively associated with inter-daily stability (B = −0.001, *p* < 0.05). The interaction remained significant after conducting post-hoc sensitivity analyses controlling for age (B = −0.002, *p* < 0.05; see Supplemental Table 1).

### Aim 2: Longitudinal Models

Table 3 presents the hierarchical multiple regression results across the three models.

**Table 3:** Aim 2 Regression Results

Hierarchical Multiple Regression						
	*Dependent variable:*					
	Time 2 Depression (BDI)					
	(1)	(2)	(3)	(4)	(5)	(6)
RA	0.657 (1.445)	0.386 (1.444)				
IV			−0.857 (1.566)	−0.883 (1.569)		
IS					0.152 (1.888)	−0.446 (1.890)
BAS	−0.089 (0.057)	−0.088 (0.057)	−0.090 (0.057)	−0.091 (0.057)	−0.091 (0.058)	−0.093 (0.057)
PDS	0.140 (0.142)	0.114 (0.142)	0.140 (0.142)	0.142 (0.142)	0.142 (0.143)	0.108 (0.143)
BDI.1	0.561^**^ (0.039)	0.560^**^ (0.039)	0.560^**^ (0.039)	0.562^**^ (0.039)	0.562^**^ (0.039)	0.564^**^ (0.039)
Sex: Female	−0.502 (0.765)	−0.563 (0.762)	−0.471 (0.763)	−0.490 (0.765)	−0.492 (0.777)	−0.591 (0.772)
Race^a^: Other	−0.853 (1.037)	−0.901 (1.032)	−0.880 (1.038)	−0.850 (1.042)	−0.861 (1.045)	−0.928 (1.037)
Race^a^: White	−1.371 (0.721)	−1.407 (0.717)	−1.384 (0.722)	−1.372(0.724)	−1.370 (0.730)	−1.499^*^ (0.726)
RA X BAS		−0.520^*^ **(0.258)**				
IV X BAS				0.124 (0.301)		
IS X BAS						−0.821^*^ **(0.346)**
Constant	7.320^**^ (0.775)	7.337^**^ (0.771)	7.309^**^ (0.773)	7.302^**^ (0.774)	7.314^**^ (0.790)	7.371^**^ (0.784)
Observations	294	294	294	294	292	292
R^2^	0.446	0.454	0.446	0.447	0.445	0.456
Adjusted R^2^	0.433	0.439	0.433	0.431	0.431	0.440
Residual Std. Error	5.267 (df = 286)	5.239 (df = 285)	5.266 (df = 286)	5.273 (df = 285)	5.287 (df = 284)	5.244 (df = 283)
F Statistic	32.919^**^ (df = 7; 286)	29.617^**^ (df = 8; 285)	32.943^**^ (df = 7; 286)	28.762^**^ (df = 8; 285)	32.510^**^ (df = 7; 284)	29.615^**^ (df=8;283)

*BAS* behavioral activation system scale, *IS* inter-daily stability, *IV* intra-daily variability, *PDS* pubertal development scale, *RA* relative amplitude

*p < 0.05. **p < 0.01.

^a^Reference level for Race: Black/African American

Bold values indicate significant interactions

#### Relative amplitude

**Fig. 2:**
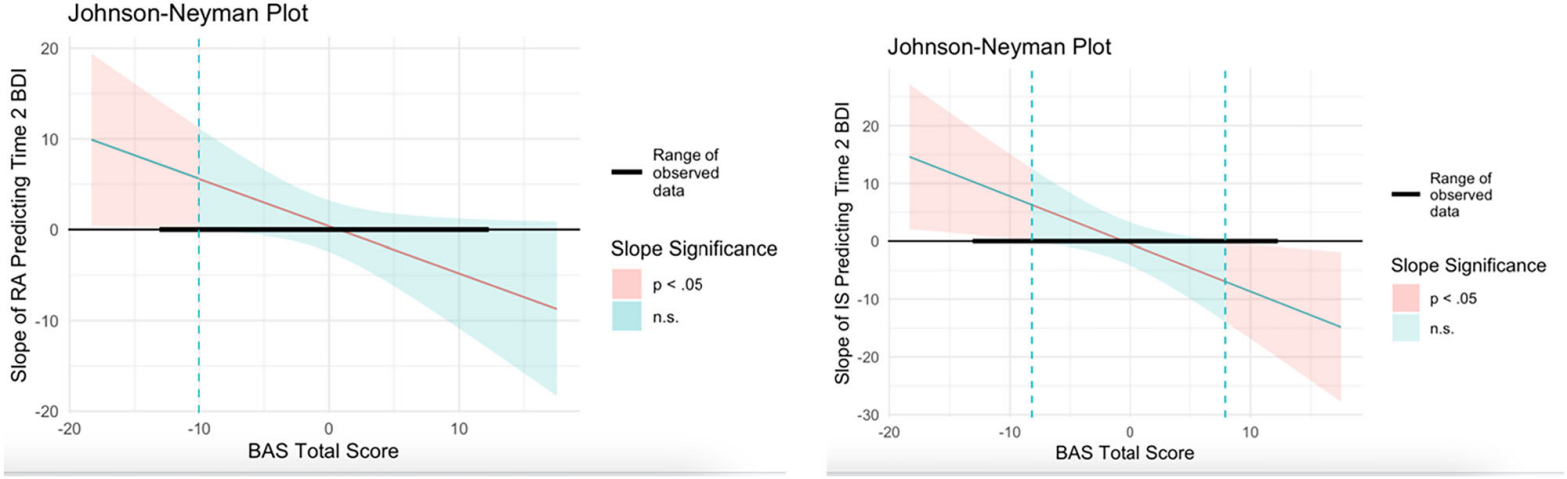
Aim 2 Interactions. The graph on the left panel represents the interaction between relative amplitude and BAS reward on Time 2 BDI whereas the graph on the right panel represents the interaction between inter-daily stability and BAS reward on Time 2 BDI. Predictor and moderator variables were centered for analyses

The first step of the model accounted for 43.26% of the variance in Time 2 depression. Relative amplitude and trait reward sensitivity were not significantly associated with Time 2 depression. In the second step of the model, the inclusion of the interaction term did not significantly increase the variance accounted for in Time 2 depression. The main effects of relative amplitude and trait reward sensitivity remained nonsignificant. However, the interaction effect was significant (B = −0.520; *p* < 0.05) (Fig. 2). Per the Johnson-Neyman Procedure, the range of observed values of trait reward sensitivity was −12.91 to 12.09, and the slope of relative amplitude was significant (*p* < 0.05) when trait reward sensitivity values were outside the interval of −10.05 to 67.40, suggesting that the interaction is only interpretable in the lower bound of the data, but not the higher bound. At very low levels of trait reward sensitivity, relative amplitude was significantly and positively associated with Time 2 depression (B = 5.61, *p* < 0.05). The interaction remained significant after conducting post-hoc sensitivity analyses controlling for age (B = −0.52, *p* < 0.05; see Supplemental Table 2).

#### Intra-daily variability

The first step of the model accounted for 43.28% of the variance in Time 2 depression. Intra-daily variability and trait reward sensitvity were not significantly associated with Time 2 depression. In the second step of the model, the inclusion of the interaction term did not significantly increase the variance accounted for in Time 2 depression. The main effects of intra-daily variability and trait reward sensitivity remained nonsignificant. The interaction effect between intra-daily variability and trait reward sensitivity on Time 2 depression also was not significant.

#### Inter-daily stability

The first step of the model accounted for 43.12% of the variance explained in Time 2 depression. Neither inter-daily stability nor trait reward sensitvity were significantly associated with Time 2 depression. The second step of the model, which included the interaction term, significantly increased the variance accounted for in Time 2 depression by 0.91%. In this step of the model, the main effects of inter-daily stability and trait reward sensitvity remained nonsignificant. However, the interaction effect between inter-daily stability and trait reward sensitvity on Time 2 depression was significant (B = −0.821, *p* < 0.05), suggesting a cross-over interaction (Fig. 2). Per the Johnson-Neyman Procedure, the range of observed values of trait reward sensitvity was −12.91 to 12.09, and the slope of inter-daily stability was significant (*p* < 0.05) when trait reward sensitvity values were outside the interval of −8.18 to 7.91. At very low levels of trait reward sensitivity, inter-daily stability was significantly and positively associated with Time 2 depression (B = 6.27, *p* < 0.05), whereas, at very high levels of trait reward sensitvity, inter-daily stability was significantly and negatively associated with Time 2 depression (B = −6.94, *p* < 0.05). The interaction remained significant after conducting post-hoc sensitivity analyses controlling for age (B = −0.82 *p* < 0.05; see Supplemental Table 2).

## Discussion

Extant work suggests bidirectional links of the reward and circadian systems in relation to mood disorders (Alloy et al., 2015). However, less is known about the associations between the reward and circadian systems preceding onset of a mood disorder, specifically during adolescence. Given the substantial effect pubertal development has on both of these systems, the current study first examined concurrent associations between trait reward sensitivity and rest-activity rhythms, specifically non-parametric circadian indices, and the moderating effect of pubertal development. Findings suggest that trait reward sensitivity is differentially related with circadian indices of rhythm robustness and stability at varying levels of pubertal maturation. Second, the study examined prospective associations between circadian indices and depressive symptoms and the moderating impact of trait reward sensitivity. Findings suggest that circadian indices of rhythm robustness and stability were differentially predictive of depressive symptoms at varying levels of trait reward sensitivity. Together, these findings emphasize the importance of considering both pubertal development and trait reward sensitivity when interpreting the effects of circadian dysregulation on adolescent mental health.

In Aim 1, trait reward sensitivity was not independently associated with any of the circadian indices (i.e., relative amplitude, intra-daily variability, inter-daily stability). This was contrary to predictions. However, when considered in conjunction with pubertal development, trait reward sensitivity, as indicated by BAS, was significantly associated with both relative amplitude and inter-daily stability, controlling for age and other covariates. Consistent with predictions, there was a negative association between trait reward sensitivity and relative amplitude for adolescents later in puberty; however, there was a positive association between trait reward sensitivity and relative amplitude for those who were earlier in puberty. The same pattern emerged for the interaction between trait reward sensitivity and pubertal development on inter-daily stability. Although the significant associations observed in later stages of puberty were consistent with expectations, the significant associations observed in earlier stages of puberty appeared as an exploratory pattern. To the effect that the reward sensitivity interaction with pubertal maturation on relative amplitude and inter-daily stability held even when controlling for age indicates the specificity of the effect to pubertal development.

Taken together, patterns of circadian instability (i.e., low relative amplitude and inter-daily stability) often observed in at risk populations for mood disorders such as bipolar spectrum disorders (e.g., Alloy et al., 2012; Murray et al., 2020; McCarthy et al., 2022), were associated with high trait reward sensitivity among a community sample of adolescents who were more pubertally mature (i.e., approximately Tanner stage 5). These findings suggest that the link between reward sensitivity and circadian instability may become more salient as adolescents progress through puberty (e.g., Hasler et al., 2012). Recent genetic research suggests a link between the same index of rhythm robustness, a flattened (i.e., lower) relative amplitude, and mood variability (Ferguson et al., 2018; Shi et al., 2008), potentially emphasizing the clinical utility of detecting this pattern of circadian dysregulation early on prior to onset of mood disorders.

Contrary to hypotheses, trait reward sensitivity was not significantly associated with the fragmentation of the rest-activity cycle, as indicated by intra-daily variability, in its joint effect with pubertal development. It is possible that trait reward sensitivity explains more variance in the strength and consistency of circadian rhythms *between* days as opposed to variability *within* a 24-hr period, emphasizing the difference in time scale of the two indices (inter-daily stability vs. intra-daily variability). Further work should examine whether the timing of sleep midpoint potentially explains the null findings involving intra-daily variability.

For Aim 2, there were no significant main effects between the three circadian indices and changes in Time 2 depressive symptoms. However, both relative amplitude and inter-daily stability were significantly predictive of changes in Time 2 depressive symptoms when considered in conjunction with trait reward sensitivity, controlling for Time 1 depressive symptoms, pubertal development, age, and other covariates. Consistent with the reward-circadian model (reviewed in Alloy et al., 2015), less circadian rhythm stability between days, as indicated by a lower inter-daily stability, was predictive of greater depressive symptoms among those who exhibited high trait reward sensitivity; however, the inverse relationship was true for those who endorsed low trait reward sensitivity. Specifically, for those with low trait reward sensitivity, a more stable circadian rhythm between days was predictive of greater depressive symptoms at Time 2. This finding potentially highlights the importance of coupling to zeitgebers and how synchronization may be differentially related to depressive symptoms depending on an individual’s level of trait reward sensitivity.

Among those who endorsed a very low trait reward sensitivity, a more robust circadian rhythm, as indicated by relative amplitude, was predictive of greater Time 2 depressive symptoms, but the inverse of this association was not supported. Contrary to predictions, the fragmentation of the rhythm, as indicated by intra-daily variability, also was not predictive of depressive symptoms in conjunction with trait reward sensitivity. Although initially surprising, the observed, positive simple slopes at low levels of trait reward sensitivity potentially delineates a key difference in circadian patterns between adolescents at risk for unipolar depression versus bipolar depression. Specifically, people at risk for and diagnosed with a major depressive disorder often endorse both a blunted reward sensitivity (Alloy et al., 2016) and a more sedentary lifestyle (Liu et al., 2016; Zhai et al., 2015), potentially leading to a more stable circadian rhythm across days. If both daytime and nighttime activity are low, the calculation of relative amplitude may yield a moderately high index (Blume et al., 2016). An alternative explanation for this exploratory pattern is potentially forced or maladaptive entrainment. Considering adolescents often experience circadian misalignment due to regimented social demands such as school (Smith et al., 2023), this could lead to a mismatch between internal and external demands, resulting in increased depressive symptoms. Additional longitudinal research is needed to further understand the generalizability of this finding. Overall, these findings illustrate that the three non-parametric indices may differentially relate to mood vulnerability, which potentially highlights the importance of synchronization, as in inter-daily stability, within the realm of depression (Ehlers et al., 1988).

The current study offers several strengths. First, non-parametric circadian rhythm analysis was conducted to provide a more granular inspection of rest-activity rhythms compared to traditional metrics. Other actigraphy analyses often impose a cosine waveform shape on the data, which adds statistical limitations by automatically fitting the data to a pre-assumed shape; however, non-parametric circadian rhythm analysis allows for a more accurate inspection between sleep and wake intervals, which is particularly relevant among populations who may deviate from a normative rest-activity cycle (Blume et al., 2016; Van Someren et al., 1999). Second, participant recruitment was based specifically on trait reward sensitivity, which allowed us to study a sample of adolescents who were representative of the full dimension of reward sensitivity. Third, the current study included a community, adolescent sample, which allowed for a developmental perspective when examining potential patterns of risk that may explain the increased vulnerability for mood dysregulation experienced in this period.

There are, however, a few limitations worthy of note. First, we collected continuous actigraphy data for an average of one week. Although this is standard in much of the literature, collecting data for three weeks would provide greater reliability in determining rest-activity patterns across both weekdays and weekends. Second, there were small effect sizes for Aim I interactions despite significance. However, small effect sizes still can translate into having a meaningful impact on the population level, especially if findings are generalizable. Future research should explore whether stronger effects emerge in clinical populations. Finally, due to project design, the current study was unable to examine models inclusive of hypomanic or manic symptoms. As a next step, future research should examine these models in prediction of hypo/mania to better discern differential effects between circadian dysregulation and the full spectrum of mood symptomatology. Additional longitudinal research is needed to assess whether these patterns of circadian instability are, indeed, predictive of episode onset to clarify an at-risk framework (Alloy et al., 2015).

## Conclusion

Adolescence often is accompanied by increased mood dysregulation and mood episode onset. Therefore, it is crucial to determine early patterns that may provide an explanation for the observed increased risk. Several biological changes occur during adolescence including shifts to the reward and circadian systems. However, less is known about how these systems interact in relation to mood preceding onset of mood disorders. Often, symptoms such as internal mood states (i.e., depression) are difficult to objectively detect on their own, encouraging researchers to examine observable aspects of behavior. Specifically, an adolescent’s rest-activity rhythm represents an overt, behavioral aspect that may be more accessible to track in relation to mood vulnerability. Thus, the current study examined associations between trait reward sensitivity, pubertal development, rest-activity rhythms, and depressive symptoms in an adolescent sample. As pubertal development increased, high trait reward sensitivity was associated with lower relative amplitude and inter-daily stability, a commonly observed pattern of dysregulation in mood disorders. Among those with high trait reward sensitivity, lower inter-daily stability predicted greater depressive symptoms, whereas the opposite was true for those with low trait reward sensitivity. Together, these findings highlight the importance of considering both pubertal development and reward processes when attempting to understand the impact of circadian dysregulation on adolescent mood. Moreover, the integration of sleep and circadian health in the education system would provide an avenue for increased awareness given the tangible and positive effects of practicing appropriate sleep hygiene. Although longitudinal research is needed to refine these associations and their relevance to risk, the current study represents a necessary step in identifying developmental patterns involving rest-activity rhythms in adolescence.

## Supplementary information

Supplemental materials
