# Supplementary material for: Reward Sensitivity, Pubertal Development, and Circadian Rhythms: A Window into Adolescent Risk for Depressive Symptoms
Source: J Youth Adolesc. 2025 Aug 20;54(10):2467–80. doi: 10.1007/s10964-025-02224-6 (PMC12370276; doi:10.1007/s10964-025-02224-6)

Sensitivity Analyses

Supplemental Table 1. Aim 1 Regression Results Controlling for Age

| **Hierarchical Multiple Regression** | | | | | | |
| --- | --- | --- | --- | --- | --- | --- |
|  | | | | | | |
|  | *Dependent variable:* | | | | | |
|  |  | | | | | |
|  | RA | | IV | | IS | |
|  | (1) | (2) | (3) | (4) | (5) | (6) |
|  | | | | | | |
| BAS | -0.002 (0.002) | -0.002 (0.002) | 0.002 (0.002) | 0.002 (0.002) | -0.003^*^ (0.002) | -0.003^*^ (0.002) |
| PDS | 0.004 (0.006) | 0.004 (0.006) | -0.001 (0.006) | -0.001 (0.006) | 0.005 (0.005) | 0.005 (0.005) |
| Sex: Female | 0.044 (0.030) | 0.046 (0.029) | -0.007 (0.027) | -0.007 (0.027) | 0.040^*^ (0.023) | 0.042^*^ (0.023) |
| Race^+^: Other | 0.002 (0.040) | 0.007 (0.039) | -0.027 (0.037) | -0.026 (0.037) | 0.020 (0.031) | 0.024 (0.030) |
| Race^+^: White | 0.027 (0.028) | 0.023 (0.027) | -0.025 (0.025) | -0.026 (0.026) | 0.034 (0.021) | 0.031 (0.021) |
| Age | -0.006 (0.013) | -0.005 (0.013) | -0.004 (0.012) | -0.004 (0.012) | -0.016 (0.010) | -0.016 (0.010) |
| BAS X PDS |  | **-0.003^***^ (0.001)** |  | -0.0005 (0.001) |  | **-0.002^***^ (0.001)** |
| Constant | 0.787^***^ (0.207) | 0.777^***^ (0.205) | 0.852^***^ (0.190) | 0.850^***^ (0.190) | 0.576^***^ (0.159) | 0.568^***^ (0.157) |
|  | | | | | | |
| Observations | 320 | 320 | 320 | 320 | 318 | 318 |
| R^2^ | 0.023 | 0.049 | 0.008 | 0.009 | 0.049 | 0.076 |
| Adjusted R^2^ | 0.004 | 0.028 | -0.011 | -0.013 | 0.031 | 0.055 |
| Residual Std. Error | 0.214 (df = 313) | 0.212 (df = 312) | 0.196 (df = 313) | 0.196 (df = 312) | 0.164 (df = 311) | 0.162 (df = 310) |
| F Statistic | 1.231 (df = 6; 313) | 2.297^**^ (df = 7; 312) | 0.412 (df = 6; 313) | 0.405 (df = 7; 312) | 2.666^**^ (df = 6; 311) | 3.640^***^ (df = 7; 310) |
|  | | | | | | |

*Note.* BAS = Behavioral Activation System Scale; IS = Inter-daily stability; IV = Intra-daily variability; PDS = Pubertal Development Scale; RA = Relative amplitude. ^+^ Reference level for Race: Black/African American.

* *p* < .05. ** *p* < .01. ******* *p* < .001

Supplemental Table 2. Aim 2 Regression Results Controlling for Age

| **Hierarchical Multiple Regression** | | | | | |
| --- | --- | --- | --- | --- | --- |
|  | | | | | |
|  | *Dependent variable:* | | | | |
|  |  | | | | |
|  | Time 2 BDI | | | | |
|  | (1) | (2) | (3) | (4) | (5) |
|  | | | | | |
| RA | 0.645 (1.448) | 0.374 (1.447) |  |  |  |
| IV |  |  | -0.853 (1.568) | -0.879 (1.572) |  |
| IS |  |  |  |  | -0.500 (1.901) |
| BAS | -0.090 (0.057) | -0.089 (0.057) | -0.090 (0.057) | -0.092 (0.057) | -0.095 (0.057) |
| PDS | 0.158 (0.159) | 0.132 (0.159) | 0.159 (0.159) | 0.161 (0.159) | 0.130 (0.159) |
| BDI.1 | 0.562^**^ (0.039) | 0.560^**^ (0.039) | 0.560^**^ (0.039) | 0.562^**^ (0.039) | 0.565^**^ (0.039) |
| Sex: Female | -0.546 (0.786) | -0.609 (0.782) | -0.518 (0.784) | -0.536 (0.786) | -0.640 (0.790) |
| Race^+^: Other | -0.860 (1.039) | -0.909 (1.034) | -0.888 (1.040) | -0.858 (1.045) | -0.934 (1.039) |
| Race^+^: White | -1.361 (0.723) | -1.396 (0.720) | -1.373^*^(0.724) | -1.361 (0.726) | -1.484^*^ (0.729) |
| Age | -0.090 (0.352) | -0.094 (0.350) | -0.093 (0.352) | -0.093 (0.352) | -0.107 (0.354) |
| RA X BAS |  | **-0.520^*^ (0.259)** |  |  |  |
| IV X BAS |  |  |  | 0.124 (0.302) |  |
| IS X BAS |  |  |  |  | **-0.823^*^ (0.347)** |
| Constant | 8.689 (5.416) | 8.762 (5.387) | 8.727 (5.414) | 8.711 (5.422) | 8.999 (5.427) |
|  | | | | | |
| Observations | 294 | 294 | 294 | 294 | 292 |
| R^2^ | 0.446 | 0.454 | 0.447 | 0.447 | 0.456 |
| Adjusted R^2^ | 0.431 | 0.437 | 0.431 | 0.429 | 0.438 |
| Residual Std. Error | 5.275 (df = 285) | 5.247 (df = 284) | 5.274 (df = 285) | 5.282 (df = 284) | 5.253 (df = 282) |
| F Statistic | 28.718^**^ (df = 8; 285) | 26.249^**^ (df = 9; 284) | 28.740^**^ (df = 8; 285) | 25.491^**^ (df = 9; 284) | 26.250^**^ (df = 9; 282) |
|  | | | | | |

*Note.* BAS = Behavioral Activation System Scale; IS = Inter-daily stability; IV = Intra-daily variability; PDS = Pubertal Development Scale;

RA = Relative amplitude. ^+^ Reference level for Race: Black/African American.

* *p* < .05. ** *p* < .01. ******* *p* < .001

Sex-Stratified Analyses

Supplemental Table 3. Aim 1 Regression Results Within Male Subgroup

| **Hierarchical Multiple Regression** | | | | | | |
| --- | --- | --- | --- | --- | --- | --- |
|  | | | | | | |
|  | *Dependent variable:* | | | | | |
|  |  | | | | | |
|  | RA | | IV | | IS | |
|  | (1) | (2) | (3) | (4) | (5) | (6) |
|  | | | | | | |
| BAS | 0.003 (0.004) | 0.003 (0.004) | 0.002 (0.003) | 0.002 (0.003) | 0.002 (0.003) | 0.002 (0.003) |
| PDS | 0.011 (0.008) | 0.008 (0.008) | -0.001 (0.008) | -0.002 (0.008) | 0.009 (0.006) | 0.006 (0.006) |
| Race^+^: Other | -0.028 (0.065) | -0.029 (0.064) | -0.046 (0.060) | -0.046 (0.060) | 0.002 (0.049) | 0.001 (0.048) |
| Race^+^: White | 0.024 (0.049) | 0.027 (0.049) | -0.053 (0.045) | -0.052 (0.046) | 0.006 (0.037) | 0.009 (0.037) |
| BAS X PDS |  | -0.002 (0.002) |  | -0.001 (0.001) |  | -0.002 (0.001) |
| Constant | 0.698^**^ (0.041) | 0.695^**^ (0.041) | 0.816^**^ (0.038) | 0.814^**^ (0.038) | 0.347^**^ (0.031) | 0.343^**^ (0.031) |
|  | | | | | | |
| Observations | 136 | 136 | 136 | 136 | 135 | 135 |
| R^2^ | 0.030 | 0.043 | 0.015 | 0.018 | 0.020 | 0.041 |
| Adjusted R^2^ | 0.0005 | 0.006 | -0.015 | -0.020 | -0.010 | 0.004 |
| Residual Std. Error | 0.227 (df = 131) | 0.227 (df = 130) | 0.212 (df = 131) | 0.212 (df = 130) | 0.170 (df = 130) | 0.169 (df = 129) |
| F Statistic | 1.016 (df = 4; 131) | 1.168 (df = 5; 130) | 0.495 (df = 4; 131) | 0.482 (df = 5; 130) | 0.671 (df = 4; 130) | 1.099 (df = 5; 129) |
|  | | | | | | |

*Note.* BAS = Behavioral Activation System Scale; IS = Inter-daily stability; IV = Intra-daily variability; PDS = Pubertal Development Scale; RA = Relative amplitude. ^+^ Reference level for Race: Black/African American.

* *p* < .05. ** *p* < .01. ******* *p* < .001

Supplemental Table 4. Aim 2 Regression Results Within Male Subgroup

| **Hierarchical Multiple Regression** | | | | | | |
| --- | --- | --- | --- | --- | --- | --- |
|  | | | | | | |
|  | *Dependent variable:* | | | | | |
|  |  | | | | | |
|  | Time 2 BDI | | | | | |
|  | (1) | (2) | (3) | (4) | (5) | (6) |
|  | | | | | | |
| RA | -0.537 (2.065) | -1.431 (2.077) |  |  |  |  |
| IV |  |  | -4.092 (2.191) | -4.090 (2.199) |  |  |
| IS |  |  |  |  | -1.730 (2.813) | -2.362 (2.777) |
| BAS | 0.017 (0.088) | 0.021 (0.087) | 0.020 (0.086) | 0.015 (0.088) | 0.019 (0.088) | 0.010 (0.086) |
| PDS | 0.037 (0.203) | -0.006 (0.201) | 0.037 (0.199) | 0.038 (0.200) | 0.053 (0.204) | -0.022 (0.203) |
| BDI.1 | 0.686^**^ (0.078) | 0.668^**^ (0.077) | 0.669^**^ (0.077) | 0.671^**^ (0.077) | 0.691^**^ (0.078) | 0.683^**^ (0.077) |
| Race^+^: Other | -1.851 (1.566) | -1.757 (1.543) | -2.107 (1.549) | -2.052 (1.560) | -1.931 (1.583) | -1.907 (1.555) |
| Race^+^: White | -2.756^*^ (1.184) | -2.612^*^ (1.168) | -3.000^*^ (1.174) | -2.973^*^ (1.180) | -2.892^*^ (1.209) | -2.786^*^ (1.189) |
| RA X BAS |  | **-0.782^*^ (0.367)** |  |  |  |  |
| IV X BAS |  |  |  | 0.182 (0.421) |  |  |
| IS X BAS |  |  |  |  |  | **-1.188^*^ (0.521)** |
| Constant | 6.812^**^ (0.999) | 6.773^**^ (0.985) | 6.970^**^ (0.989) | 6.938^**^ (0.995) | 6.920^**^ (1.025) | 6.893^**^ (1.007) |
|  | | | | | | |
| Observations | 124 | 124 | 124 | 124 | 123 | 123 |
| R^2^ | 0.425 | 0.447 | 0.441 | 0.442 | 0.426 | 0.451 |
| Adjusted R^2^ | 0.396 | 0.413 | 0.413 | 0.409 | 0.396 | 0.417 |
| Residual Std. Error | 5.170 (df = 117) | 5.093 (df = 116) | 5.096 (df = 117) | 5.114 (df = 116) | 5.181 (df = 116) | 5.089 (df = 115) |
| F Statistic | 14.421^**^ (df = 6; 117) | 13.387^**^ (df = 7; 116) | 15.412^**^ (df = 6; 117) | 13.146^**^ (df = 7; 116) | 14.348^**^ (df = 6; 116) | 13.487^**^ (df = 7; 115) |
|  | | | | | | |

*Note.* BAS = Behavioral Activation System Scale; IS = Inter-daily stability; IV = Intra-daily variability; PDS = Pubertal Development Scale; RA = Relative amplitude. ^+^ Reference level for Race: Black/African American.

* *p* < .05. ** *p* < .01. ******* *p* < .001

Supplemental Table 5. Aim 1 Regression Results Within Female Subgroup

| **Hierarchical Multiple Regression** | | | | | | |
| --- | --- | --- | --- | --- | --- | --- |
|  | | | | | | |
|  | *Dependent variable:* | | | | | |
|  |  | | | | | |
|  | RA | | IV | | IS | |
|  | (1) | (2) | (3) | (4) | (5) | (6) |
|  | | | | | | |
| BAS | **-0.005^*^ (0.003)** | -0.006^*^ (0.003) | 0.002 (0.003) | 0.002 (0.003) | **-0.006^**^ (0.002)** | -0.006^**^ (0.002) |
| PDS | -0.005 (0.007) | -0.004 (0.007) | -0.001 (0.007) | -0.001 (0.007) | -0.003 (0.006) | -0.002 (0.006) |
| Race^+^: Other | 0.030 (0.051) | 0.037 (0.050) | -0.015 (0.047) | -0.014 (0.047) | 0.035 (0.040) | 0.038 (0.040) |
| Race^+^: White | 0.016 (0.033) | 0.012 (0.033) | -0.008 (0.031) | -0.009 (0.031) | 0.039 (0.026) | 0.037 (0.026) |
| BAS X PDS |  | -0.002 (0.001) |  | -0.0004 (0.001) |  | -0.001 (0.001) |
| Constant | 0.747^**^ (0.026) | 0.749^**^ (0.026) | 0.774^***^(0.024) | 0.774^**^ (0.024) | 0.373^**^ (0.021) | 0.374^**^ (0.021) |
|  | | | | | | |
| Observations | 184 | 184 | 184 | 184 | 183 | 183 |
| R^2^ | 0.028 | 0.043 | 0.004 | 0.005 | 0.062 | 0.068 |
| Adjusted R^2^ | 0.007 | 0.017 | -0.018 | -0.023 | 0.041 | 0.041 |
| Residual Std. Error | 0.202 (df = 179) | 0.201 (df = 178) | 0.186 (df = 179) | 0.186 (df = 178) | 0.158 (df = 178) | 0.158 (df = 177) |
| F Statistic | 1.307 (df = 4; 179) | 1.615 (df = 5; 178) | 0.181 (df = 4; 179) | 0.163 (df = 5; 178) | 2.944^*^ (df = 4; 178) | 2.572^*^ (df = 5; 177) |
|  | | | | | | |

*Note.* BAS = Behavioral Activation System Scale; IS = Inter-daily stability; IV = Intra-daily variability; PDS = Pubertal Development Scale; RA = Relative amplitude. ^+^ Reference level for Race: Black/African American.

* *p* < .05. ** *p* < .01. ******* *p* < .001

Supplemental Table 6. Aim 2 Regression Results Within Female Subgroup

| **Hierarchical Multiple Regression** | | | | | | |
| --- | --- | --- | --- | --- | --- | --- |
|  | | | | | | |
|  | *Dependent variable:* | | | | | |
|  |  | | | | | |
|  | Time 2 BDI | | | | | |
|  | (1) | (2) | (3) | (4) | (5) | (6) |
|  | | | | | | |
| RA | 1.296 (2.032) | 1.319 (2.039) |  |  |  |  |
| IV |  |  | 2.267 (2.175) | 2.326 (2.189) |  |  |
| IS |  |  |  |  | 0.586 (2.570) | 0.424 (2.591) |
| BAS | -0.180^*^ (0.076) | -0.180^*^ (0.077) | -0.195^*^ (0.075) | -0.195^*^ (0.075) | -0.187^*^ (0.078) | -0.187^*^ (0.078) |
| PDS | 0.280 (0.208) | 0.275 (0.209) | 0.277 (0.207) | 0.274 (0.208) | 0.278 (0.209) | 0.269 (0.210) |
| BDI.1 | 0.518^**^(0.045) | 0.518^**^ (0.045) | 0.523^**^ (0.045) | 0.520^**^ (0.046) | 0.519^**^ (0.045) | 0.520^**^ (0.046) |
| Race: Other | -0.113 (1.398) | -0.143 (1.405) | -0.042 (1.394) | -0.066 (1.400) | -0.092 (1.404) | -0.127 (1.408) |
| Race: White | -0.603 (0.912) | -0.625 (0.917) | -0.535 (0.909) | -0.547 (0.912) | -0.599 (0.921) | -0.670 (0.931) |
| RA X BAS |  | -0.110 (0.370) |  |  |  |  |
| IV X BAS |  |  |  | -0.139 (0.428) |  |  |
| IS X BAS |  |  |  |  |  | -0.271 (0.473) |
| Constant | 7.375^**^ (0.741) | 7.369^**^ (0.744) | 7.320^**^ (0.738) | 7.341^**^ (0.743) | 7.358^**^ (0.746) | 7.348^**^ (0.747) |
|  | | | | | | |
| Observations | 170 | 170 | 170 | 170 | 169 | 169 |
| R^2^ | 0.468 | 0.469 | 0.471 | 0.471 | 0.466 | 0.467 |
| Adjusted R^2^ | 0.449 | 0.446 | 0.451 | 0.448 | 0.446 | 0.444 |
| Residual Std. Error | 5.271 (df = 163) | 5.285 (df = 162) | 5.260 (df = 163) | 5.274 (df = 162) | 5.292 (df = 162) | 5.303 (df = 161) |
| F Statistic | 23.942^**^ (df = 6; 163) | 20.420^**^ (df = 7; 162) | 24.155^**^ (df = 6; 163) | 20.605^**^ (df = 7; 162) | 23.531^**^ (df = 6; 162) | 20.133^**^ (df = 7; 161) |
|  | | | | | | |

*Note.* BAS = Behavioral Activation System Scale; IS = Inter-daily stability; IV = Intra-daily variability; PDS = Pubertal Development Scale; RA = Relative amplitude. ^+^ Reference level for Race: Black/African American.

* *p* < .05. ** *p* < .01. ******* *p* < .001

Exploratory Analyses of Mapping Pubertal Development Scale (PDS) Scores to Approximate Tanner Stages

Supplemental Figure 1. Supplemental Figure 2.


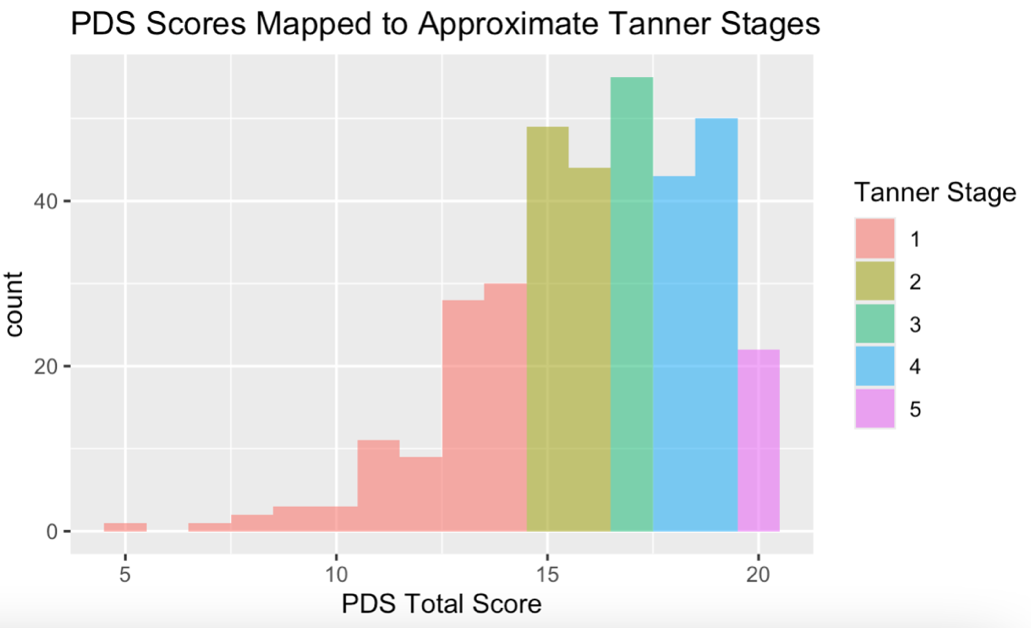

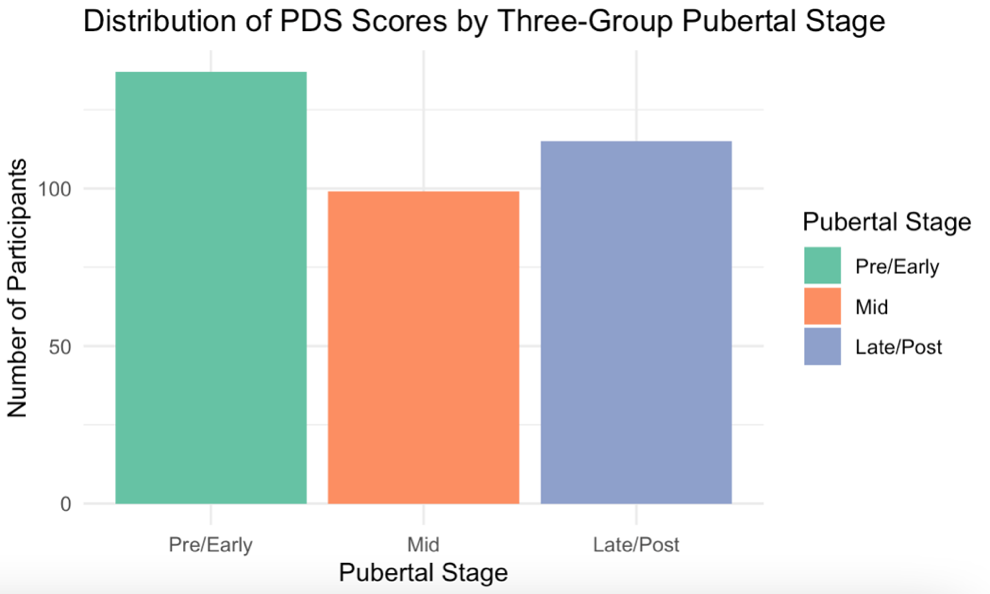

Supplement: Supplementary file 1 — Supplemental materials [file 10964_2025_2224_MOESM1_ESM.docx]
